# Expression analysis of microRNA as prognostic biomarkers in colorectal cancer

**DOI:** 10.18632/oncotarget.14175

**Published:** 2016-12-26

**Authors:** Jie Yang, Dongling Ma, Andrew Fesler, Haiyan Zhai, Apisri Leamniramit, Wenzhe Li, Song Wu, Jingfang Ju

**Affiliations:** ^1^ Translational Research Laboratory, Department of Pathology, Stony Brook University, Stony Brook, NY 11794, USA; ^2^ The NMS Laboratory, Stony Brook University, Stony Brook, NY, 11794, USA; ^3^ Department of Applied Mathematics, Stony Brook University, Stony Brook, NY, 11794, USA; ^4^ Department of Family, Population and Preventive Medicine, Stony Brook, NY 11794, USA

**Keywords:** colorectal cancer, prognosis, microRNA, chemotherapy

## Abstract

microRNA (miRNA) based biomarkers have unique advantages due to their critical regulatory function, superior stability, and relatively small number compared to mRNAs. A number of miRNAs play key roles in colon cancer stem cell chemoresistance and have clinical potential as prognostic biomarkers. The purpose of this study is to systematically validate the prognostic potential of miRNAs in colorectal cancer. In this study, we validated the prognostic potential of a panel of miRNAs using 205 stage II, III, and IV colorectal cancer specimens by qRT-PCR analysis. We cross validated our results using The Cancer Genome Atlas (TCGA) database. Many of the miRNAs we investigated have been functionally validated to be important in contributing to chemoresistance to 5-fluorouracil (5-FU) based chemotherapy. We determined that miR-16 is the most consistent miRNA for expression normalization in colorectal cancer. We have validated several miRNAs (miR-15b, miR-215, miR-145, miR-192, let-7g) that are significantly associated with progression free survival (PFS) and/or overall survival (OS) of colorectal cancer patients independent of tumor stage and age at diagnosis. These 5 miRNAs are significantly associated with OS of colorectal cancer even after tumor location (left side vs. right side) is adjusted for. Furthermore, the prognostic value of let-7g for overall survival was independently validated using the RNA-Seq results from TCGA colorectal cancer database. These results, taken together, establish a solid foundation towards miRNA based precision management of colorectal cancer.

## INTRODUCTION

Colorectal cancer is one of the leading causes of cancer related death in the United States with more than 50,000 deaths every year [1]. The current 5-year survival rate for stage II colorectal cancer patients is between 70-80%. Surgery is the standard treatment option for stage II colorectal cancer. However, about 30% of stage II patients will have relapse and there is no reliable biomarker to determine which patients are at high risk and should be managed with adjuvant chemotherapy. As for advanced stage III and IV colorectal cancer patients, despite years of effort, there is still a lack of highly reliable prognostic biomarkers to determine which patients will benefit from chemotherapy. In both early and advanced stages of colorectal cancers, there is clearly an unmet need for biomarkers for better clinical management.

Recently, it has been recognized that epigenetic changes play a key role in tumorigenesis and resistance to chemotherapy [2, 3]. Resistance to 5-fluorouracil (5-FU) based chemotherapy is the major reason for failures in treating advanced colorectal cancer. Colorectal cancer cells are highly heterogeneous, chemotherapy can be quite effective in eliminating most of the rapidly proliferating cancer cells. However, a small population of slow proliferating, cancer stem cells, is highly resistant and leads to recurrence [4]. Although the mechanism of chemoresistance to 5-Fluorouracil (5-FU) is complex and is often associated with elevated 5-FU target enzyme thymidylate synthase (TS, TYMS) [5, 6], recent studies have shown that epigenetic alterations such as non-coding miRNAs are major contributors to resistance mechanisms to 5-FU. miRNAs regulate acute changes in protein synthesis at the post-transcriptional and translational levels [3, 7–12].

miRNAs are a class of small non-coding RNAs with crucial regulatory function [13, 14]. miRNAs modulate protein expression by promoting RNA degradation, inhibiting mRNA translation, and in some cases, affecting transcription. miRNA regulation of gene expression, provides cancer cells with an advantage in response to genotoxic stress and growth condition changes. We have determined through systematic evaluation that miRNAs are highly stable in archival formalin fixed paraffin embedded (FFPE) colorectal tumor specimens [15]. This result provides the foundation for the investigation of miRNA based biomarkers using large deposits of archival FFPE specimens with long term clinical follow up information. We subsequently demonstrated the clinical significance of miRNAs (e.g. let-7g, miR-15a, miR-215, miR-129, miR-181b, miR-140, miR-200c) in colorectal cancer, especially for long term survival for patients treated with fluoropyrimidine based chemotherapy [9, 16–19]. Our subsequent studies demonstrated that these miRNAs have direct functional significance in colorectal cancer by regulating key targets such as thymidylate synthase (TYMS, TS) [20], dihydrofolate reductase (DHFR) [7], histone deacetylase [3], E2F3, and Bcl-2 [18]. Some of which are directly linked to chemoresistance in highly resistant colon cancer stem cells [12, 19].

In this study, we systematically validated the prognostic potential of candidate miRNAs in stage II, III and IV colorectal cancer. We quantified expression of a panel of 11 miRNAs (Let-7g, miR-15a, miR-15b, miR-21, miR-140, miR-143, miR-145, miR-181b, miR-192, miR-200, miR-215) selected based on their critical functions in chemoresistance and cell death in colorectal cancer as well as several profiling studies [21, 22]. We also quantified four housekeeping genes (RNU44, 5S, β-actin, miR-16) to determine the best housekeeping gene for normalizing of miRNA expression. Our results show that the best housekeeping miRNA for normalization is miR-16. We discovered several significant miRNAs (miR-15b, miR-215, miR-145, miR-192, let-7g) as prognostic biomarkers of OS that are independent of tumor stage and age at diagnosis. It has been demonstrated that the primary tumor location is an important prognostic factor in metastatic colorectal cancer [23–26]. We also analyzed the prognostic potential of these miRNAs by taking into account the primary tumor location being left side or right side. Our results show that let-7g is significantly associated with OS with independent validation using TCGA colorectal cancer datasets.

## RESULTS

### Determining the best housekeeping gene for normalization of miRNA in colorectal cancer

**Figure 1:**
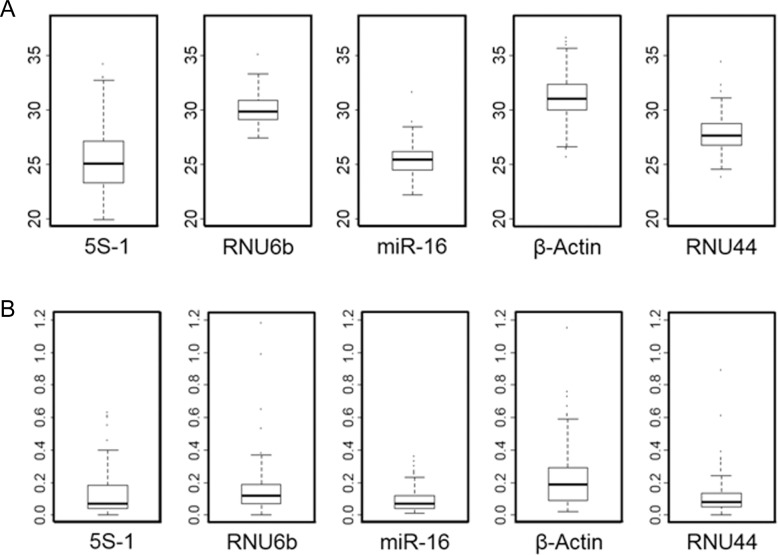
Boxplot of mean Ct. values of different housekeeping candidates **A.** Boxplot of standard deviations of the differential housekeeping gene candidates **B.**

One of the most important considerations for expression based biomarker analysis is to find and validate a true housekeeping gene for normalization. We have systematically searched the literature and selected several candidates for miRNA expression normalization in colorectal cancer. The candidates that we selected are ribosomal RNA 5S, RNU6b, β-actin, RNU44 and miR-16. Based on the expression profiles of these genes from 200 colorectal cancer samples, we show that miR-16 is the best housekeeping gene for miRNA expression analysis (Figure 1). The relative C_T_ values of gene expression were listed in Figure 1A. Our results show that miR-16 is relatively more abundant than the other housekeeping gene candidates. The standard deviation of the expression of each potential housekeeping gene are listed in Figure 1B. We have previously used RNU44 as a normalization control and RNU44 is also a good housekeeping gene with lower standard deviation than miR-16. However, due to its relative low expression levels compared to miR-16, we chose miR-16 as the best housekeeping gene for this study.

### Survival analysis

We analyzed the significance of miRNA expression with progression free survival (PFS) and overall survival (OS) based on all patients after adjusting for stage and age at diagnosis. The association results were summarized in Table 1. Our results show that low expression of Let-7g, miR-15b or miR-192 were significantly associated with better OS (estimated HRs of low expression versus high expression were 0.62, 0.26, 0.64 with p-values 0.0238, 0.0024 and 0.0406, respectively) while high expression of miR-145 was significantly associated with better OS (estimated HR=2.71 with p-value=0.0077). Low expression of miR-15b and miR-215 were also significantly associated with better PFS (estimated HRs = 0.33, 0.48 with p-values=0.0132 and 0.0235, respectively) while high expression of miR-145 was significantly associated with better PFS (estimated HR=2.50 with p-value=0.0138). In stratified analysis of stage III/IV patients, low expression of miR-21, miR-200 and miR-215 were also associated with better OS (estimated HRs=0.22, 0.44, 0.47 with p-values=0.0362, 0.0137, 0.0269, respectively) while miR-145 was not significantly associated with OS. Among advanced stage III/IV colorectal cancer patients, low expression of miR-15a was associated with worse PFS (estimated HR=2.36 with p-value=0.0081) and low expression of miR-200 and miR-215 were associated with better PFS (estimated HRs=0.49 and 0.30 with p-values=0.0194 and 0.0064, respectively). Different association results were found in stage II colorectal cancer patients: low expression of miR-143 was significantly associated with better OS and PFS (estimated HR=0.38, 0.48 with p-values=0.008, 0.0391, respectively); high expression of miR-145 and miR-181b were significantly associated with better OS and PFS (all estimated HRs>1 with p-values<0.05). In addition, low expression of miR-15b was associated with better OS in stage II patients (estimated HR=0.19 with p-value=0.0122).

**Table 1:** Estimated association between miRNA and overall survival/progression free survival after adjusting for stage, age at diagnosis based on PCR data

miRNA	Stage II/III/IV patients, HR(95% CI)	p-value	Stage III/IV patients, HR(95% CI)	p-value	Stage II patients, HR(95% CI)	p-value
**Overall survival**						
Let-7g	0.62(0.41,0.94)	0.0238	0.55(0.33,0.90)	0.0182	0.52(0.23,1.18)	0.1165
miR-15a	0.29(0.07,1.22)	0.0906	2.00(0.99,4.01)	0.0519	0.45(0.11,1.90)	0.2784
miR-15b	0.26(0.11,0.62)	0.0024	0.24(0.08,0.71)	0.0103	0.32(0.09,1.10)	0.0705
miR-21	0.37(0.13,1.03)	0.0579	0.22(0.05,0.91)	0.0362	1.61(0.85,3.08)	0.1456
miR-140	0.46(0.17,1.25)	0.128	0.62(0.31,1.23)	0.1728	1.62(0.83,3.16)	0.1535
miR-143	1.55(0.57,4.23)	0.3949	0.21(0.03,1.55)	0.1257	0.38(0.19,0.78)	0.008
miR-145	2.71(1.30,5.63)	0.0077	1.81(0.86,3.82)	0.1193	2.40(1.15,5.01)	0.0201
miR-181b	1.37(0.92,2.02)	0.1199	0.43(0.17,1.08)	0.0732	2.75(1.28,5.87)	0.0092
miR-192	0.64(0.41,0.98)	0.0406	0.49(0.29,0.84)	0.0094	2.06(0.63,6.72)	0.2293
miR-200	0.73(0.48,1.10)	0.137	0.44(0.23,0.85)	0.0137	2.08(0.95,4.54)	0.0664
miR-215	0.57(0.29,1.10)	0.0928	0.47(0.24,0.92)	0.0269	1.80(0.93,3.49)	0.0798
**Progression free survival**						
Let-7g	0.72(0.46,1.13)	0.1564	0.57(0.26,1.26)	0.1634	0.64(0.28,1.44)	0.2812
miR-15a	1.64(0.99,2.71)	0.054	2.36(1.25,4.47)	0.0081	0.61(0.31,1.22)	0.1608
miR-15b	0.33(0.14,0.79)	0.0132	0.41(0.14,1.18)	0.0985	0.19(0.05,0.69)	0.0122
miR-21	0.54(0.22,1.33)	0.1765	0.39(0.12,1.26)	0.1158	0.59(0.25,1.40)	0.2282
miR-140	1.36(0.93,1.99)	0.1089	1.55(0.92,2.59)	0.0964	1.34(0.71,2.54)	0.3639
miR-143	0.86(0.59,1.26)	0.4317	0.66(0.35,1.24)	0.1942	0.48(0.24,0.96)	0.0391
miR-145	2.50(1.21,5.19)	0.0138	0.69(0.41,1.15)	0.1519	3.47(1.22,9.88)	0.02
miR-181b	1.29(0.89,1.88)	0.1769	0.51(0.22,1.17)	0.1126	3.34(1.37,8.12)	0.0078
miR-192	0.72(0.48,1.08)	0.1104	0.64(0.39,1.05)	0.0757	2.17(0.77,6.07)	0.1414
miR-200	0.76(0.52,1.13)	0.1779	0.49(0.27,0.89)	0.0194	1.61(0.81,3.19)	0.1763
miR-215	0.48(0.25,0.91)	0.0235	0.30(0.13,0.71)	0.0064	1.68(0.91,3.11)	0.0994

### Survival analysis considering primary tumor location

In addition to the survival analysis without considering the primary tumor location, we recognized that tumor location (left vs. right) is a significant prognostic factor to be considered when stratifying patient survival in colorectal cancer [23, 24]. We further analyzed the association of PFS and OS by including the primary tumor location. Our results show that tumor location does indeed influence the association between miRNAs and patient survival (Table 2). In terms of OS, the expression of let-7g, miR-15b, miR-145, and miR-192 were significant prognostic biomarkers for stage II/III/IV colorectal cancer patient survival and miR-215 became associated with OS after further controlling for tumor location (estimated HR=0.49), but it was on the border-line (p-value=0.0499). The expression of let-7g, miR-15b, miR-21, miR-192, miR-200, and miR-215 were still significantly associated OS among stage III/IV colorectal cancer patients. The expression of miR-143, miR-145, and miR-181b were still significantly associated with OS among stage II colorectal cancer patients. For PFS association, the expression of miR-215 was still significantly associated with stages II/III/IV patients but the expression of miR-15b and miR-145 were not after adjusting for tumor location. miR-15a, miR-215 and miR-200 expression levels were still significantly associated with stage III/IV PFS. miR-143, miR-145 and miR-181b expression levels were still significantly associated with stage II PFS, but miR-15b expression level was not significantly associated with stage II PFS after considering tumor location.

**Table 2:** Estimated association between miRNA and overall survival/progression free survival after adjusting for stage, age at diagnosis and tumor location based on PCR data (N=187)

miRNA	Stage II/III/IV patients, HR(95% CI)	p-value	Stage III/IV patients, HR(95% CI)	p-value	Stage II patients, HR(95% CI)	p-value
Overall survival						
Let-7g	0.55(0.34,0.89)	0.0143	0.5(0.29,0.86)	0.0115	0.47(0.20,1.15)	0.0991
miR-15a	0.3(0.07,1.26)	0.1005	1.63(0.8,3.32)	0.1799	0.63(0.28,1.45)	0.2766
miR-15b	0.61(0.4,0.92)	0.0195	0.53(0.31,0.91)	0.0206	0.56(0.28,1.14)	0.1082
miR-21	0.34(0.1,1.13)	0.0797	0.23(0.05,0.97)	0.046	1.52(0.77,3.01)	0.2235
miR-140	0.62(0.35,1.07)	0.0859	0.58(0.29,1.18)	0.1312	1.77(0.85,3.67)	0.1264
miR-143	0.73(0.44,1.22)	0.2341	0.49(0.24,1.03)	0.0585	0.38(0.18,0.81)	0.0121
miR-145	2.13(1.02,4.47)	0.0443	0.59(0.33,1.05)	0.074	2.40(1.07,5.38)	0.034
miR-181b	2.88(0.69,11.99)	0.1452	0.37(0.13,1.04)	0.0591	2.86(1.27,6.43)	0.0109
miR-192	0.63(0.4,0.99)	0.0441	0.51(0.3,0.86)	0.0123	2.31(0.70,7.56)	0.1679
miR-200	0.66(0.42,1.02)	0.0629	0.49(0.28,0.86)	0.0123	2.25(0.97,5.22)	0.0581
miR-215	0.49(0.24,1)	0.0499	0.31(0.14,0.69)	0.0043	2.03(1.00,4.15)	0.0513
Progression free survival						
Let-7g	0.67(0.42,1.09)	0.1079	0.66(0.4,1.08)	0.1	0.60(0.25,1.44)	0.2562
miR-15a	1.48(0.87,2.52)	0.1465	1.98(1.01,3.88)	0.0459	0.58(0.27,1.22)	0.1515
miR-15b	0.76(0.51,1.14)	0.186	0.64(0.38,1.07)	0.0896	0.49(0.16,1.49)	0.207
miR-21	0.55(0.2,1.52)	0.2475	0.22(0.03,1.63)	0.1396	0.62(0.26,1.46)	0.2709
miR-140	0.65(0.38,1.09)	0.1019	1.59(0.93,2.73)	0.0917	1.47(0.74,2.93)	0.2738
miR-143	0.79(0.53,1.19)	0.256	0.62(0.37,1.03)	0.0669	0.47(0.23,0.94)	0.0323
miR-145	1.89(0.91,3.94)	0.0893	0.6(0.34,1.04)	0.0662	2.63(1.23,5.63)	0.0125
miR-181b	2.17(0.67,7.02)	0.1981	0.47(0.19,1.19)	0.1104	2.45(1.11,5.41)	0.0261
miR-192	0.71(0.46,1.08)	0.1079	0.64(0.38,1.06)	0.0847	2.98(0.72,12.41)	0.1332
miR-200	0.7(0.46,1.06)	0.0911	0.57(0.33,0.96)	0.0337	1.67(0.81,3.46)	0.1654
miR-215	0.43(0.22,0.84)	0.0136	0.27(0.12,0.61)	0.0015	1.93(0.98,3.82)	0.0584

### Survival analysis using TCGA data

**Figure 2:**
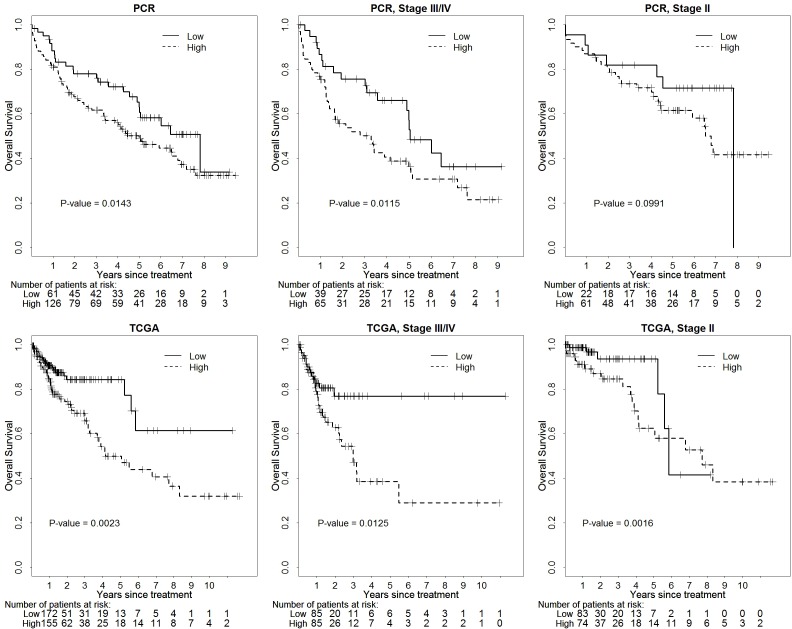
Kaplan-Meier survival analysis examining the association between let-7g expression in colorectal tumors with overall survival based on Stony Brook University patient cohort and TCGA colorectal cancer patient cohort

To further validate the results, we used our own data as the experimental set, and we analyzed the miRNA expression with patient survival by using TCGA colorectal cancer data as a validation set. Our results show that TCGA data is highly consistent with our results generated from patient samples at Stony Brook University Medical Center (Table 3). The expression levels of let-7g, miR-15a, miR-200 and miR-181 were significantly associated with OS of stage II/III/IV patients before and after considering tumor location. The representative Kaplan-Meier OS survival curves of let-7g based on our data and TCGA data for different patient groups are shown in Figure 2. The expression levels of let-7g, miR-21, miR140, miR143, miR-181, miR-192, and miR-215 are significantly associated with stage III/IV patient’s OS. Low expression of miR-15b was associated with better OS in stage III/IV patients, but this did not achieve statistical significance after considering tumor location. Among stage II patients, findings from our data were also confirmed by TCGA data while additional miRNAs were shown to be significantly associated with stage II patients’ OS such as let-7g, miR-15a, miR-140, miR-192 and miR-200.

**Table 3:** Estimated association between miRNA and overall survival after adjusting for stage, age at diagnosis based on TCGA colorectal cancer data

miRNA	Stage II/III/IV patients, HR(95% CI)	p-value	Stage III/IV patients, HR(95% CI)	p-value	Stage II patients, HR(95% CI)	p-value
Without further adjusting for tumor location						
Let-7g	0.48(0.28,0.80)	0.0049	0.48(0.26,0.90)	0.0227	0.51(0.20,1.30)	0.1559
miR-15a	1.92(1.13, 3.26)	0.0151	0.52(0.26,1.03)	0.0603	3.46(1.13,10.56)	0.0291
miR-15b	0.75(0.43,1.31)	0.3169	0.31(0.12,0.81)	0.0163	2.53(1.03,6.20)	0.0429
miR-21	1.70(0.73, 3.97)	0.221	2.49(1.11,5.56)	0.0264	0.25(0.11,0.57)	0.0012
miR-140	0.51(0.20, 1.30)	0.1579	2.09(1.07,4.09)	0.0318	0.43(0.19,0.99)	0.0463
miR-143	0.58(0.32,1.07)	0.0812	1.88(1.07,3.32)	0.029	0.34(0.14,0.79)	0.0118
miR-145	0.64(0.38,1.07)	0.0902	2.04(0.85,4.85)	0.1089	0.50(0.22,1.12)	0.0924
miR-181b_1	0.57(0.35, 0.95)	0.0312	0.57(0.32,1.00)	0.0509	0.45(0.18,1.11)	0.0834
miR_181b_2	0.41(0.13,1.32)	0.1365	0.55(0.31,0.97)	0.0407	1.69(0.75,3.82)	0.2071
miR-192	0.71(0.45, 1.14)	0.1558	0.43(0.24,0.78)	0.0057	9.82(1.31,73.48)	0.0262
miR-215	0.50(0.25, 1.01)	0.0539	0.43(0.18,1.02)	0.055	2.27(1.01,5.12)	0.0472
miR-181	0.52(0.32, 0.84)	0.0068	0.43(0.24,0.76)	0.0037	0.26(0.06,1.21)	0.0852
miR-200	2.49(1.06,5.84)	0.0355	3.25(0.79,13.39)	0.1028	2.48(1.06,5.79)	0.0361
With further adjusting for tumor location						
Let-7g	0.45(0.27,0.75)	0.0023	0.45(0.24,0.84)	0.0125	0.43(0.26,0.73)	0.0016
miR-15a	1.95(1.14,3.34)	0.0144	0.58(0.29,1.14)	0.1165	1.03(1.01,1.05)	0.0081
miR-15b	0.77(0.44,1.34)	0.3579	0.72(0.4,1.31)	0.2819	1.20(0.73,1.97)	0.4803
miR-140	1.52(0.92,2.51)	0.099	2.4(1.19,4.83)	0.0146	1.03(1.01,1.05)	0.0046
miR-143	0.6(0.33,1.1)	0.0987	2.01(1.13,3.57)	0.0175	1.26(0.77,2.08)	0.3614
miR-145	0.65(0.39,1.08)	0.0988	1.87(0.78,4.45)	0.1583	1.76(1.08,2.88)	0.023
miR-192	0.7(0.43,1.13)	0.1477	0.4(0.22,0.73)	0.0028	0.59(0.36,0.98)	0.0413
miR-21	1.92(0.69,5.35)	0.2101	1.92(1.07,3.43)	0.0281	1.03(1.01,1.05)	0.0058
miR-215	0.52(0.26,1.05)	0.0702	0.35(0.14,0.91)	0.0306	1.18(0.71,1.95)	0.5178
miR-181	0.5(0.31,0.81)	0.0047	0.42(0.23,0.75)	0.0033	1.93(1.14,3.24)	0.0135
miR-200	2.12(1,4.48)	0.0505	3.35(0.81,13.86)	0.0945	1.03(1.01,1.05)	0.0083

## DISCUSSION

In this study, we evaluated the prognostic potential of miRNAs in colorectal cancer based on 200 patient samples with clinical outcome follow up information. We have also systematically validated the housekeeping controls that can be used for miRNA expression normalization in colorectal cancer. Our findings are consistent with previous reports that RNU44 and miR-16 are two genes with the least expression variation in colorectal cancer [9, 27]. We chose miR-16 as the housekeeping miRNA for this study as it has a relatively higher level of expression than RNU44 with the best standard deviation (Figure 1). miR-16 has been shown to be the ideal housekeeping candidate in breast cancer and pancreatic cancer [28, 29]. Our results are also consistent with previous studies that miR-16 is a suitable housekeeping miRNA in colon cancer [27]. We show that miR-15a and miR-15b are significantly associated with colorectal cancer patient’s survival (Table 1 and 2). This is in contrast to CLL in which a cluster containing miR-15 and miR-16 is deleted from 13q14 [2]. There might be unique differential processing of miR-15 and miR-16 in solid tumors such as breast cancer, pancreatic cancer and colorectal cancer. As miR-15a/16-1 cluster is located in chromosome 13 while miR-15b/miR-16-2 is located in chrosomsome 3. Identification and utilization of the proper housekeeping gene is one of the most important factors for any biomarker study as the housekeeping gene used for miRNA expression normalization is often times different in unique tumor types.

There are a number of advantages to using miRNA as biomarkers. miRNAs are relatively stable in archival FFPE samples which makes them superior to degraded mRNAs [15]. This allows for large scale retrospective studies using archival FFPE specimens. In addition, their aberrant expression may be indicative of the disruption and dysregulation of multiple cellular networks.

Our studies have identified miRNAs that are significantly associated with stage II, III, and IV colorectal cancer patients. This study further validated several miRNAs (e.g. miR-21, miR-200, miR-215) with prognostic potential in colorectal cancer from our own group as well as several other reports [9, 16, 17, 21, 30]. More importantly, these are supported by our previously discovered functional significance of these miRNAs in colorectal cancer resistance and EMT. A number of these miRNAs (e.g. miR-192, miR-215) have shown to play critical roles in colorectal cancer chemoresistance by directly regulating key mRNA target expression such as thymidylate synthase and dihydrofolate reductase [7, 20]. miR-192 and miR-215 are directly regulated by the tumor suppressor gene p53 in colon cancer [7, 20]. We have also demonstrated previously that miR-181b is significantly associated with chemotherapeutic response in colorectal cancer [9, 16].

One of the novel and significant aspects of this study is to take colorectal tumor location into consideration. This has never been investigated in conjunction with the expression of miRNAs in colorectal cancer. Previous studies have shown that primary tumor location (left vs. right) is a significant prognostic factor in metastatic colorectal cancer [23]. When we include primary tumor location as a factor with miRNA expression, we show that let-7g, miR-15b, miR145, miR-200, and miR-215 are still significantly associated with patient’s OS. This is highly consistent with our previous studies that let-7g is closely associated with colon cancer chemoresponse [16]. We have also shown that miR-200 has prognostic potential in colorectal cancer, which is consistent with previous studies [9]. It is well established that miR-200 plays key roles in EMT by regulating Zeb1 expression [31].

We further cross validated the findings based on colorectal cancer patients from Stony Brook University Medical Center with colorectal cancer TCGA database containing RNA-Seq expression data for all miRNAs [32]. Based on TCGA miRNA dataset with tumor location information, we were able to show that let-7g, miR-140, miR-200, miR-192, miR-181 remained highly significant prognostic factors for metastatic colorectal cancer independent of tumor stage. Our results are also consistent with some of the miRNA based colorectal cancer biomarker studies [21, 33]. Let-7g is the most consistent prognostic biomarker between the Stony Brook University Medical Center Cohort and TCGA (Figure 2). Patients with low expression of let-7g have better survival compared to the group of patients with high expression. This is in contrast to the general notion that let-7g has a tumor suppressor role. We reason that this may be due to the fact that tumor cells in patients with lower let-7g levels may have a more rapid proliferation rate, making them more sensitive to 5-FU and oxaliplatin based DNA damaging agents used in colorectal cancer chemotherapy. This is in fact consistent with another tumor suppressive miRNA, miR-215, as patients with low expression have improved survival compared to patients with higher expression [17].

In conclusion, we systematically validated several miRNAs with clinical prognostic potential for metastatic colorectal cancer using patient cohorts from Stony Brook University Medical Center and TCGA. With cancer clinical management moving to more personalized approaches, this study provides a foundation to better prepare us to leverage these potential biomarkers to assist future clinical management of colorectal cancer.

## MATERIALS AND METHODS

### Clinical Samples

We selected 205 colorectal cancer specimens from patients who underwent surgical resection of primary tumors at the Stony Brook University Medical Center, Stony Brook, NY, USA. Patient consent forms were obtained from each patient according to institutional policies. Patient clinical information was provided by the Cancer Registry of Stony Brook University Medical Center, and the characteristics of these patients are shown in Table 4. Among these, we have 89 cases of stage II, 86 cases of stage III and 30 stage IV colorectal cancer patient archival formalin fixed paraffin embedded (FFPE) tissue specimens. Representative tissue blocks from each case were assembled from the archival collections of the Department of Pathology, and used for subsequent analysis. 200 patients had both follow-up information and miRNA expression information and 187 patients had tumor location information available.

**Table 4:** Patient population summary

Stony Brook Patients Diagnosed (1998–2013)	N=205*
Sex	Male 104 (50.73%) Female 101 (49.24%)
Age of Diagnosis	Mean- 66.50 S.D.- 13.90 Range- 28-99
Stage	II 89 (43.41%) III 86 (41.95%) IV 30 (14.63%)
Location**	Left 89 (46.84%) Right 101 (53.16%)
Chemotherapeutic Intervention	Yes 93 (45.37%) No 112 (54.63%)
Survival	<5 Years 132 (66%) >5 Years 71 (35%)

Clinical and expression data used for validation in this study were downloaded from the UCSC cancer genome browser (http://xena.ucsc.edu/), which is a set of web-based tools to display and investigate cancer genomics data and its associated clinical information [34]. Specifically, we extracted the clinical and miRNA expression data for TCGA colon adenocarcinoma. Genome-wide characterizations of the expression patterns of mRNA and miRNA of these samples have been reported previously [32]. For the clinical data, the survival information for 431 subjects is available. The miRNA and mRNA expression was measured using HiSeq platform. There are 331 subjects that have both survival and miRNA expression data and 327 subjects that have tumor location, survival and miRNA expression data.

### RNA isolation and qRT-PCR analysis

FFPE specimens are deparaffinized using xylene and ethanol washes as previously described. Samples were digested with protease to recover total RNA. RNA were purified using a rapid glass-fiber filter methodology from Thermo Fisher Scientific (Ambion RecoverAll™ Total Nucleic Acid Isolation Kit for FFPE) that includes an on-filter DNase treatment to remove contaminated genomic DNA. Purified RNA samples are eluted with nuclease free water for cDNA synthesis and quantitative RT-PCR analysis.

The reverse transcription of miRNAs to cDNAs were conducted using TaqMan miRNA RT kit from Thermo Fisher Scientific (Life Technologes) by combining primers for different miRNAs using 40 ng of purified total RNA. Multiplex qRT-PCR reactions were performed using the Thermo Fisher Scientific (Applied Biosystems Inc.) 7900HT Fast Real Time PCR Detection System with 95°C for 10 min, then 40 cycles of 95°C for 15 seconds, 60°C for 60 seconds. miRNA level was analyzed with its specific primers and internal housekeeping control miR-16. Fluorescent signals from each sample were collected at the endpoint of every cycle, and the expression level of each unique miRNA was calculated by ΔΔ*C*_T_ values based on the internal controls, normalized to control group and plotted as relative value (RQ).

### Data analysis

Overall survival (OS) was defined as the time from the date of diagnosis to the last follow-up date or the date of death whichever occurred first. Progression free survival (PFS) was defined as the time from the date of diagnosis to the last follow-up date or the date of death or the date of recurrence whichever occurred first, and only being alive at the last follow-up date was considered as censored. Normalized expression levels were defined using the 2^−ΔΔCT^ method with miR-16 used as housekeeping control gene. Each miRNA’s expression was dichotomized into low and high expression using a cutoff value in the expression level which gave the smallest p-value to test if the specific miRNA expression was associated with survival outcomes based on multivariable Cox proportional hazard model after adjusting for age at diagnosis and cancer stage. Estimated hazard ratios and their 95% confidence intervals for each miRNA between low expression and high expression were reported for both OS and PFS. Stratified analysis for early stage (stage II) and advanced stage (stage III, IV) were performed. TCGA data were analyzed similarly. Statistical analysis was performed using SAS 9.3 (SAS Institute, Inc., Cary, NC) and Kaplan-Meier curves were estimated using R i386 3.3.0. Statistical significance was set at 0.05.
